# Novel method combining endoscopic band ligation and clipping for hemostasis of colonic diverticular bleeding

**DOI:** 10.1055/a-2109-1195

**Published:** 2023-07-13

**Authors:** Noritaka Ozawa, Kenji Yamazaki, Haruka Koizumi, Kiichi Otani, Kosuke Hasegawa, Shogo Shimizu, Masahito Shimizu

**Affiliations:** 1 Department of Gastroenterology, Gifu Prefectural General Medical Center, Gifu, Japan; 2 Department of Gastroenterology, Gifu University School of Medicine, Gifu, Japan

Several endoscopic modalities are used to treat colonic diverticular bleeding (CDB), a predominant lower gastrointestinal bleeding type 1 2 . Most modalities involve clipping or endoscopic band ligation (EBL), which is preferred for preventing early rebleeding 3 4 5 ; however, rebleeding often occurs even after EBL. Moreover, previous studies have debated whether EBL or clipping is more effective 3 4 5 . We have established a novel method combining EBL and clipping (i. e. the EBL-C method) for hemostasis of CDB.

A 49-year-old man presented with a chief complaint of bloody stools. Contrast-enhanced computed tomography revealed extravasation from the descending colonic diverticulum. We diagnosed him with CDB and performed emergency colonoscopy.

When a descending colonic diverticulum was aspirated and inverted, a visible vessel was found. Active bleeding occurred due to suction stimulation, and the diverticulum was identified as the bleeding source ( Fig. 1 ). Endoscopic hemostasis was achieved by the EBL-C method ( Video 1 ).

**Fig. 1:**
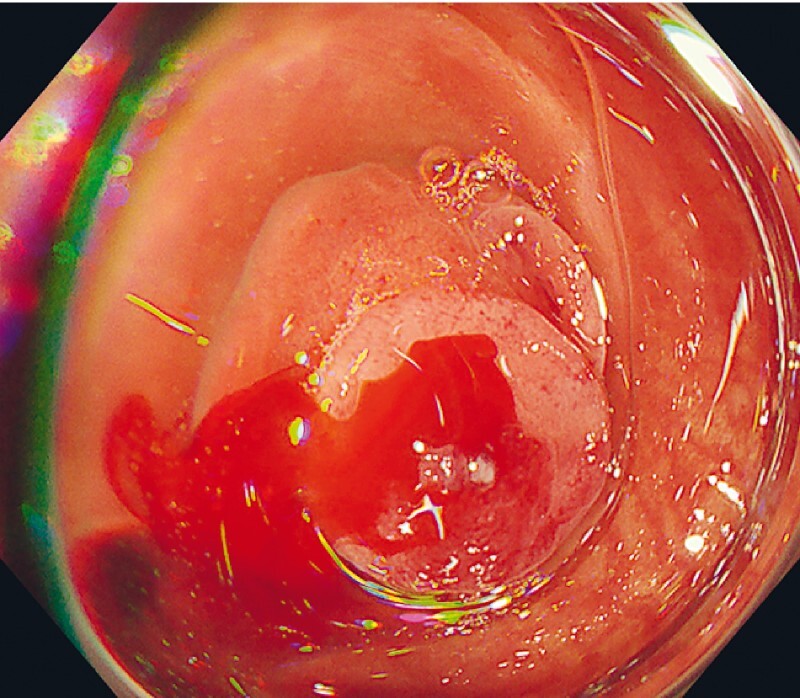
Active bleeding from the diverticulum was confirmed with suction.

**Video 1** Endoscopic hemostasis of colonic diverticular bleeding by combining endoscopic band ligation and clipping.


Endoscopic clipping was performed by placing a hemostatic clip (SureClip; Micro-Tech Co., Nanjing, China) onto the responsible vessel within the diverticulum. The endoscope was removed, and an EBL device (Sumitomo Bakelite Co., Ltd., Tokyo, Japan) was attached to its tip. It was then reinserted and directed toward the diverticulum marked with a clip. The diverticulum was aspirated with the clip into the EBL device and the elastic O-band was released ( Fig. 2 ). No rebleeding occurred after treatment ( Fig. 3 ).

**Fig. 2:**
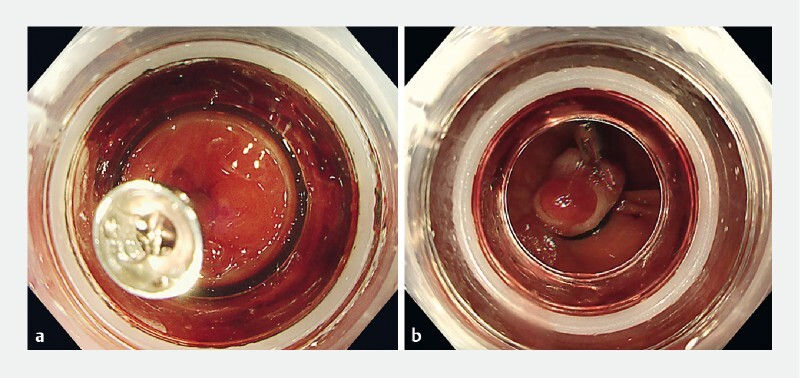
Endoscopic band ligation for hemostasis. **a** Aspiration of the diverticulum with the clip into the endoscopic band ligation device. **b** The O-band was released, and the procedure was completed.

**Fig. 3:**
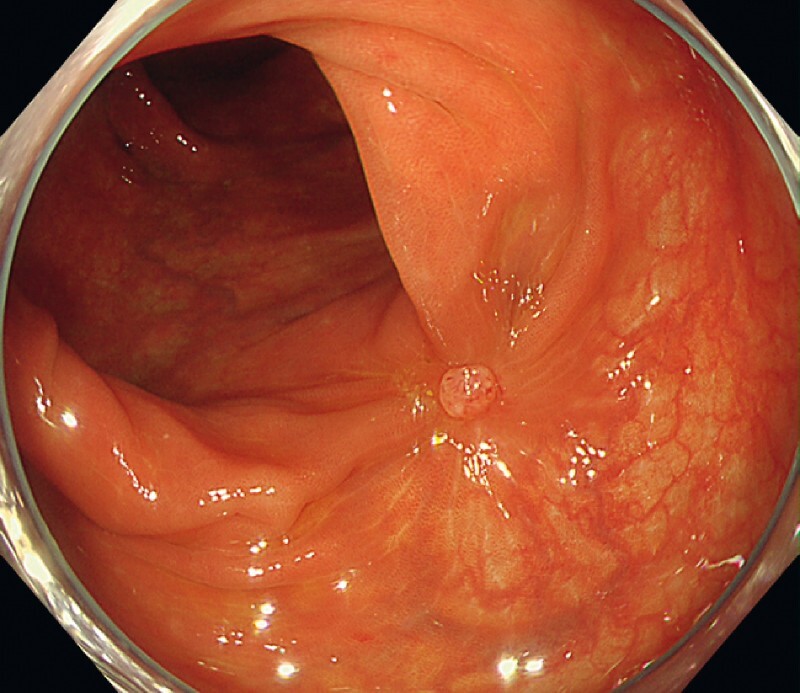
The endoscopic band ligation–clipping scar 1 month after the procedure.

Clipping involves grasping the exposed blood vessel part, whereas EBL involves grasping the root of the blood vessel. Therefore, the novel EBL-C combination treatment provided a stronger and more reliable hemostatic effect than that of each treatment alone ( Fig. 4 ). The clip served as a marker during endoscopic reinsertion with the attached EBL device. Clipping hemostasis prevented bleeding during endoscopic reinsertion, thereby preventing visual field disorientation due to blood pools. In cases where clipping is insufficient, EBL can have a synergistic hemostatic effect.

**Fig. 4:**
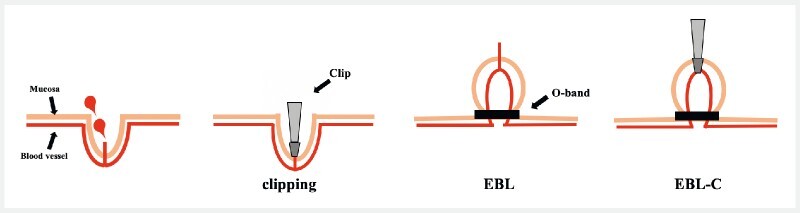
The endoscopic band ligation–clipping (EBL-C) method. Clipping: grasping the exposed part of the blood vessel. EBL: grasping the base of the blood vessel. EBL-C: combining EBL and clipping.

This is the first reported case describing the EBL-C method for hemostasis of CDB. The EBL-C method could be a simple alternative for CDB treatment with less risk of rebleeding compared with EBL or clipping alone, if confirmed in a large case series.

Endoscopy_UCTN_Code_TTT_1AO_2AD

## Notes

Endoscopy E-Videos

https://eref.thieme.de/e-videos
*E-Videos* is an open access online section of the journal *Endoscopy* , reporting on interesting cases and new techniques in gastroenterological endoscopy. All papers include a high-quality video and are published with a Creative Commons CC-BY license. Endoscopy E-Videos qualify for HINARI discounts and waivers and eligibility is automatically checked during the submission process. We grant 100% waivers to articles whose corresponding authors are based in Group A countries and 50% waivers to those who are based in Group B countries as classified by Research4Life (see: https://www.research4life.org/access/eligibility/ ). This section has its own submission website at https://mc.manuscriptcentral.com/e-videos
